# Elucidation of rutin’s role in inducing caspase-dependent apoptosis via HPV-E6 and E7 down-regulation in cervical cancer HeLa cells

**DOI:** 10.1042/BSR20210670

**Published:** 2021-06-21

**Authors:** Pratibha Pandey, Fahad Khan, Mohd Farhan, Asif Jafri

**Affiliations:** 1 Department of Biotechnology, Noida Institute of Engineering and Technology, Greater Noida 201306, India; 2 Department of Basic Sciences, King Faisal University, Al Ahsa 31982, Kingdom of Saudi Arabia; 3 Department of Zoology, University of Lucknow, Lucknow 226007, India

**Keywords:** Cervical cancer, E6 and E7, HeLa, molecular docking, p53 and pRb, Rutin

## Abstract

Over the recent few years rutin has gained wider attention in exhibiting inhibitory potential against several oncotargets for inducing apoptotic and antiproliferative activity in several human cancer cells. Several deregulated signaling pathways are implicated in cancer pathogenesis. Therefore we have inclined our research towards exploring the anticancerous efficacy of a very potent phytocompound for modulating the incontinent expression of these two crucial E6 and E7 oncogenes. Further, inhibitory efficacy of rutin against human papillomavirus (HPV)-E6 and E7 oncoproteins in cervical cancer has not been elucidated yet. This research addresses the growth inhibitory efficacy of rutin against E6 and E7 oncoproteins in HeLa cells, which is known to inactivate several tumor suppressor proteins such as p53 and pRB. Rutin treatment exhibited reduced cell viability with increased cell accumulation in G_0_/G_1_ phase of cell cycle in HeLa cell lines. Additionally, rutin treatment has also led to down-regulation of E6 and E7 expression associated with an increased expression of p53 and pRB levels. This has further resulted in enhanced Bax expression and decreased Bcl-2 expression releasing cytochrome *c* into cytosol followed by caspase cascade activation with cleavage of caspase-3, caspase-8 and caspase-9. Further, *in silico* studies have also supported our *in vitro* findings by exhibiting significant binding energy against selected target oncoproteins. Therefore, our research findings might recommend rutin as one of the potent drug candidate in cervical cancer management via targeting two crucial oncoproteins associated with viral progression.

## Introduction

Human papillomavirus (HPV)-induced cervical cancer has been recognized as one of the major health issues among women and accounts for high mortality rate due to its poor prognosis and late diagnosis [1,2]. Both initial establishment and final progression of this cancer have been fully associated with the constitutive expression of two major oncogenes including E6 and E7. HPV-infected cells exhibit co-expression of E7 and E6, which promote persistent proliferative signaling [3,4]. Numerous therapeutic approaches have utilized these two potential biomarkers (E6 and E7 oncogenes) of cervical cancer for removing the abnormally growing malignant cells [5]. E6 and E7 are recognized as key oncoplayers driving whole HPV-mediated cervical carcinogenesis by the establishment of six potential hallmarks of cancer [6]. Thus, targeting E7 and E6 could ensure cervical cancer cell eradication through eliminating few or all of the major cancer hallmarks including uncontrolled cell proliferation, invasion, angiogenesis, uncontrolled telomerase activity, metastasis, apoptosis evasion and deregulation of growth suppressors. Earlier literatures have also proven that cancerous cells undergo apoptosis (senesce) in the absence of E7 and E6 activity, thus providing a strong evidence for the association of E6 and E7 for HPV-mediated cervical cancer [7,8].

Several therapeutics including vaccines, genome-editing techniques have shown remarkable efficiency in bringing down HPV-infected cervical cancer cells via targeting E6 and E7 expression [9,10]. Prophylactic vaccines are only effective in the early phases and cannot prevent the progression of cervical cancer in the case of late diagnosis [11]. Therefore researchers are focusing on developing other therapies such as targeting of E7 and E6 oncogenes which could further suppress metastasis of cervical cancer [12]. The E7 and E6 oncoproteins are found to interact specifically with specific tumor suppressor proteins such as pRb and p53 [13]. Both p53 and pRb plays pivotal role in negatively regulating the cell cycle and inhibits the G_0_–G_1_ and G_1_–S phase transitions. These interactions of E6 and E7 with tumor suppressor proteins play significant roles in bringing cell immortality [14].

Various conventional therapies utilized in chemoprevention poses numerous delimitations such as considerable toxicity, low efficacy and high cost [15,16]. Thus, the development of potent multitargeted agents for the attenuation of dysregulated signaling in cancer is gaining wider attention globally [17–19]. In recent years, natural or plant products have been recognized as promising drug candidate for the treatment and prevention of several carcinomas [20]. Nowadays, phytotherapeutic approaches are relying on the usage of natural compounds for the treatment of various carcinomas [21–23]. Many types of anticancer drugs utilized in modern chemotherapies are either directly produced from natural sources or their modified versions such as vincristine, docetaxel podophyllotoxin, paclitaxel, vinblastine and camptothecin [24–26]. Moreover, various plant extracts or phytocompounds have exhibited significant anticancerous potential in cervical cancer cells both *in vitro* and *in vivo* [27,28]. Rutin (rutoside) is a plant-derived flavonoid which is widely distributed in fruits, vegetables, asparagus, oranges, apricots, buckwheat, cherries, apples, grapes, plums and tea. Rutin has been reported to exhibit prodigious anticancerous potential via modulating numerous oncosuppressive/oncogenic signaling pathways and targeting autophagic, inflammatory, angiogenic and apoptotic signaling mediators [29,30]. Mechanism behind the inhibitory potential of rutin against E6 and E7 expression in cervical cancer cells has not yet been explored in detail. Thus, the present study is focused towards exploring the anticancerous potential of rutin via modulating E6 and E7 mRNA expression in HeLa cancer cells.

## Materials and methods

### Chemicals

Rutin hydrate, Hoechst 33342, Mito Tracker (Red CMX Ros) fluorescent dye, propidium iodide (PI), and 2′,7′-dichlorodihydrofluorescein diacetate (DCFH-DA) were procured from Sigma (St. Louis, MO, U.S.A.). Fetal bovine serum (FBS), Dulbecco’s modified Eagle’s medium (DMEM), 3-(4,5-dimethylthiazol-2-yl)-2,5-diphenyl tetrazolium bromide (MTT) and other reagents and chemicals were procured from HiMedia India, Ltd. (Mumbai, India). Caspase-3 and -9 activity assay kits were purchased from BioVision, U.S.A.

### Cell culture

HPV18^+^ human cervical cancer HeLa cell lines were obtained from National Centre for Cell Science (NCCS), Pune, India. HeLa cancer cells were grown in DMEM supplemented with 10% FBS (heat-inactivated) and 1% antibiotic–antimycotic solution, amphotericin B and streptomycin (Himedia India, Ltd., Mumbai, India) in controlled atmosphere having 5% CO_2_ at 37°C.

### Investigation of cell viability by MTT assay

Inhibitory effect of rutin on cervical cancer HeLa cells was analyzed by MTT assay as reported in previous studies [30]. In brief, HeLa cells were incubated for attachment in an incubator for 24 h (at 37°C) and then eventually treated with rutin (0, 40, 80, 120, 160 and 200 µM) for additional 24 h. Then, each well was supplemented with 10 μl of MTT (5 mg/ml) dye and incubated at 37°C for 4 h. Formazan or purple-colored precipitates were dissolved in DMSO (100 μl) with gentle shaking. Absorbance was assessed at 490 nm on microplate reader (Bio-Rad, U.S.A.) after complete dissolution and cell survival was evaluated as percentage over the untreated control.

### Investigation of nuclear morphology by DAPI staining

Apoptotic potential of rutin on HeLa cells was observed by fluorescent nuclear dye 4′,6-diamidino-2-phenylindole (DAPI) as per the protocol reported in our previous studies [31]. In brief, 5 × 10^4^ HeLa cancer cells/well (seeded in six-well plate) were treated with varied doses of rutin (40–200 µM) for 24 h and then fixed with paraformaldehyde (3.5%) and consequently permeabilized with 0.05% (v/v) Triton X-100. Morphological changes in nuclei (fluorescent) were observed under inverted fluorescence microscope (FLoid Cell Imaging Station, Thermo Fisher Scientific, U.S.A.).

### Investigation of caspase-3 activities in rutin-treated HeLa cancer cells

Caspase activities in rutin-treated HeLa cancer cells were estimated by Caspase-3 Colorimetric Assay Kits (BioVision, U.S.A.). In short, both control (untreated) and rutin-treated HeLa (3 × 10^6^) cancer cells were lysed in chilled cell lysis buffer and left incubated for 10 min on ice. Cell lysate was centrifuged (at 10000×***g***) for 1 min and obtained supernatant was kept on ice for instant assay. Fifty microliters of lysate was then aliquoted into 96-well plate with 50 μl of reaction buffer containing 10 mM DTT. DEVD-pNA (5 μl) substrate was then added into each well and left incubated for 1 h at 37°C. Absorbance was recorded at 405 nm on a microtiter plate reader. Percent increase in caspase-3 activity was determined by comparing the result of treated cells with untreated cells (level of uninduced control).

### Investigation of the effect of caspases (caspase-3, caspase-8 and caspase-9) inhibitors

In order to characterize rutin cytotoxicity, HeLa cancer cells were pretreated with 50 μM of respective caspase inhibitors (Z-DEVD-FMK, Z-IETD-FMK and Z-LEHD-FMK) for 2 h and then eventually treated with rutin at selective doses (40–200 µM) for 24 h. Lastly, cell viability was evaluated by using MTT assay as described in our previously published research report.

### Investigation of mitochondrial membrane potential in rutin-treated HeLa cells

Mitochondria-specific Mito Tracker (Red CMX Ros) fluorescent dye was used to monitor the variation in mitochondrial membrane potential (MMP) as described by Farooqui et al., 2017 [30]. In short, cells (seeded in a 12-well plate) were treated with several doses of rutin (80–320 μg/ml). After 12 h of treatment, HeLa cancer cells were washed with phosphate buffer saline (PBS; twice) and then exposed to 3.5% of paraformaldehyde (for cell fixation process) at 37°C for 15 min. Finally, treated cells were stained with Mito Tracker Red (25 μg/ml) dye to observe changes in MMP under fluorescence microscope (FLoid Cell Imaging Station, Thermo Fisher Scientific, U.S.A.).

### Determination of intracellular reactive oxygen species level

DCFH-DA method was utilized to evaluate reactive oxygen species (ROS) generation in rutin-treated HeLa cancer cells [30]. In brief, 1.5 × 10^4^ HeLa cancer cells/well (seeded in a 12-well plate) were incubated for 24 h at 37°C. After exposure of rutin (40–200 µM) to HeLa cancer cells for 12 h, 10 µM of DCFH-DA was added to the cells and incubated for 30 min at 37°C. Images were taken after washing excessive DCFH-DA under an inverted fluorescence microscope (Nikon ECLIPSE Ti-S, Japan). Quantitative estimation of ROS level in treated cells was done by incubating rutin-treated cells with DCFH-DA (10 µM) for 30 min at 37°C. Fluorescence intensity was recorded by multiwall microplate reader (Synergy H1 Hybrid Multi-Mode Microplate Reader, BioTek, U.S.A.) at emission wavelength (528 nm) and excitation wavelength (485 nm). Values were expressed as percentage of fluorescence intensity relative to control.

### Investigation of N-acetyl-l-cysteine efficacy

N-acetyl-l-cysteine (NAC; ROS inhibitor) was used to validate the intracellular ROS generation in rutin-treated HeLa cervical cancer cells. In brief, HeLa cancer cells were pretreated with NAC (10 mM) for 2 h followed by rutin treatment for 12 h. Cells were washed with PBS, stained with DCFH-DA (10 mM) and incubated for 30 min at 37°C. Fluorescence intensity was recorded by multiwall microplate reader at emission wavelength (528 nm) and excitation wavelength (485 nm). In order to further explore the correlation of ROS generation with apoptosis in rutin-treated HeLa cancer cells, we investigated the effect of rutin on HeLa cells in presence of 10 mM NAC by employing MTT assay.

### Investigation of cell cycle arrest in rutin-treated HeLa cancer cells

HeLa cells were seeded in 6-well plate (3 × 10^5^) and incubated overnight for adherence. Rutin-treated cells were then trypsinized, centrifuged (500×***g***) for 5 min, resuspended in PBS solution and then fixed in ethanol (70% ice cold) at 4°C for 4 h. Afterwards, cells were washed two times with PBS and treated with 0.1 mg/ml RNase A at 37°C for 30 min. Lastly, cells were stained in dark with DNA staining solution containing 0.1% (v/v) Triton X-100, 0.025 mg/ml PI for 30 min. Flourescent cells were analyzed by flow cytometry with FACSAriaII Flow Cytometer (Becton Dickinson, U.S.A.) and obtained data were further analyzed by Flow Jo 7.6.2 (TreeStar, U.S.A.) [32].

### Real-time PCR analysis

To examine whether rutin treatment exhibits any effects on transcription of anti-apoptotic or pro-apoptotic genes in HeLa cells, the mRNA expression of selected genes in DMSO control or rutin-treated cells was compared. HeLa cells were seeded and left incubated for 24 h. Adherent cells were then treated with DMSO control or rutin for 24 h. Afterwards, cells were washed with PBS (5 µl) and trypsinized. Obtained cell suspension was then centrifuged at 350×***g*** (for 3 min) and remaining cell pellet was resuspended in ice-cold PBS (800 μl). Again cell suspension was centrifuged at 17000×***g*** (for 3 min) at 4°C. Obtained cell pellet was stored at −80°C for further use. Total RNA extracted from rutin-treated HeLa cells after 24 h post-treatment using TRIzol reagent (as per the manufacturer’s protocol from Invitrogen). RT-PCR was executed using SuperScript III One-Step RT-PCR with Platinum Taq DNA polymerase kit (12574-018; Invitrogen). Relative expression of both control and treated sample was normalized to β-actin mRNA and evaluated by 2^(ΔΔ*C*_t_)^ method. Target mRNA expression data was evaluated by 2−[(*C*_t_ of the gene of interest) − (*C*_t_ of internal control)], wherein *C*_t_ means threshold cycle for every transcript. Selected primers used in the present study are listed as follows:

**Table:** 

Gene	Forward primer	Reverse primer
*Bax*	5′-AAGAAGCTGAGCGAGTGT-3′	5′-GGAGGAAGTCCAATGTC-3′
*Bcl-2*	5′-TCCATGTCTTTGGACAACCA-3′	5′-CTCCACCAGTGTTCCCATCT-3′
*Cyclin D1*	5′-ATGTGTGCAGAAGGAGGTCC-3′	5′-CCTTCATCTTAGAGGCCACG-3′
*CDK4*	5′-CAGTGTACAAGGCCCGTGATC-3′	5′-ACGAACTGTGCTGATGGGAAG-3′
*p53*	5′-TTGGGAGTAGATGGAGCCT- 3′	5′-AGAGGCAAGGAAAGGTGATA- 3′
*E6*	5′-GCCAGAAACCGTTGAATCC-3′	5′-AGTCTTTCCTGTCGTGCTCG-3′
*E7*	5′-GCATGGACCTAAGGCAACA-3′	5′-CTCGTCGGGCTGGTAAAT-3′
*pRB*	5′-GTTATCAATACCACCAGGGAG-3′	5′-AAATCTGAAACACTATAAAGCC-3′
*β-actin*	5′-GTCTGTGATGCCCTTAGATG-3′	5′-AGCTTATGACCCGCACTTAC-3′

### *In silico* analysis by molecular docking

Structure of E6 (PDB ID: 2LJZ) and E7 (PDB ID: 2B9D) (target) associated with the progression of cervical cancer were retrieved from Protein Data Bank (PDB) database [33,34]. 3D structure of rutin (ligand) present in citrus fruits was retrieved from online database (PubChem). Ligand structure was further prepared for *in silico* analysis by removing water molecules, ions and heteroatoms. Docking was performed according to the protocol followed by Khan et al., 2019 [35]. In brief, binding conformation of ligand–protein complex was analyzed by using a scoring function on the basis of free binding energy. Lamarckian Genetic Algorithm (LGA) was selected among the several search algorithms found in suite of AutoDock for docking calculations. Auto-Dock 4.0 was used to develop the grid parameter file of Jab1 and various grid points in x, y and z-axes were 60 × 60 × 60 Å. The distance between two connecting grid points was 0.375 Å. LGA run terminated after 2500000 (number) energy evaluations with ∼27000 generations. Various parameters were selected according to the default values of the software. Further, Discovery Studio (Version 4.1.0) software was employed to analyze intermolecular interactions such as hydrophobic interactions (between target protein and ligand) and hydrogen bonds.

### Statistical analysis

Data analysis was performed using Prism software (GraphPad Prism version 7.0, GraphPad Software, Inc.). Statistical analyses were performed using one-way ANOVA. Error bars for SEM are shown. Where indicated in the figures, degrees of *P*-value significance are as follows: *P<0.01 and **P<0.001.

## Results

### Rutin reduced cell viability in HeLa cervical cancer cells

**Figure 1:**
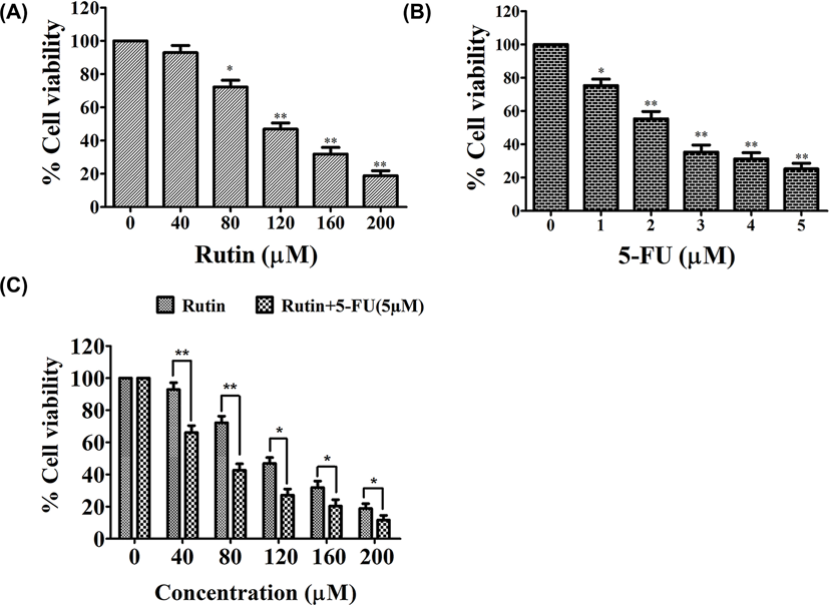
Rutin exhibited reduced viability in HeLa cancer cells after tretment with increasing doses of rutin for 24 h (**A**) MTT assay to examine cell viability. (**B**) HeLa cells were exposed to varying doses of 5-FU (1–5 μM) for 24 h. (**C**) HeLa cells were treated with combined dose of rutin (0–200 μM) + 5-FU (5 μM) to assess the synergistic effect. % cell viability (assesses by MTT assay) was normalized against untreated cells (control). SEM and mean of three independent experiments were presented: *P<0.01, **P<0.001, in comparison to related control value.

MTT assay was utilized to assess the antiproliferative efficacy of rutin on proliferation of HeLa cells. HeLa cells were treated with varied doses of rutin or 5-FU (1–5 µM) for 24 h, respectively. Figure 1 clearly exhibited significant reduction in cell viability of rutin-treated HeLa cells in a dose-dependent manner in comparison to control (Figure 1A). Origin software (Data analysis and Graphing software) was used to calculate the IC_50_ value (IC_50_ = 114.07) for selecting respective doses for further analysis. Exposure of HeLa cells to 5-FU also presented dose-dependent reduction in cell survival (Figure 1B).

The inhibitory potential of rutin on HeLa cells raised the possibility that rutin could exhibit synergistic impact with 5-FU on cell viability of HeLa cells. Figure 1C clearly showed that combined treatment of rutin and 5-FU exhibited an additional reduction in the cell viability.

### Rutin-treated HeLa cells displayed apoptosis

**Figure 2:**
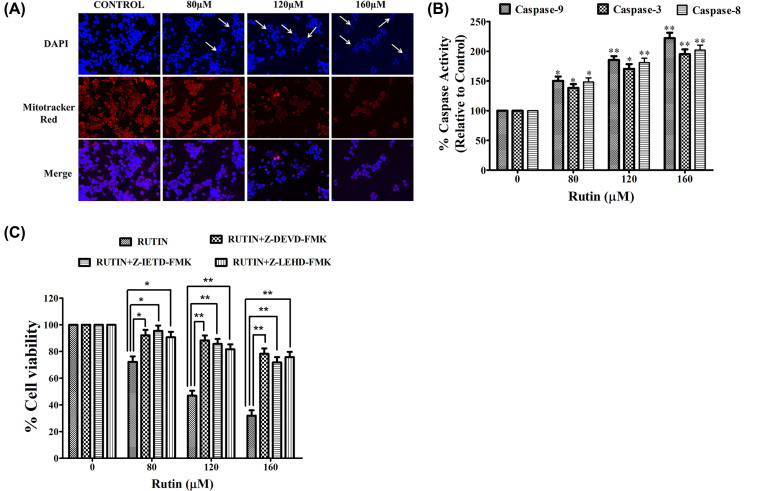
Apoptotic induction in rutin-treated HeLa cancer cells (**A**) Dose-dependent efficacy of rutin on nuclear morphology and MMP of HeLa cancer cells for 12 h, investigated by fluorescence microscopy using Mitotracker Red staining and DAPI dye, respectively. (**B**) Effects of rutin treatment on cleavage of caspases (caspase-3, caspase-8 and caspase-9) in HeLa cancer cells treated for 24 h. (**C**) HeLa cells were pretreated with three respective caspase inhibitors (Z-DEVD-FMK, Z-IETD-FMK, Z-LEHD-FMK) that suppressed their respective caspase cleavage. SEM and mean of three independent experiments were presented: *P<0.01, **P<0.001, in comparison to related control value.

To elucidate the preliminary events of apoptosis associated with nuclear morphology in rutin-treated HeLa cells, DAPI staining was carried out. HeLa cancer cells were incubated with selective doses of rutin for 24 h and apoptosis was observed using DAPI dye (Figure 2A). Apoptotic bodies were characterized with fragmented and condensed nuclei. Rutin-treated cells showed apoptotic bodies while untreated or control cells have shown no significant apoptosis. These results strongly suggested that rutin induced significant apoptosis induction via nuclear condensation in HeLa cancer cells.

### Reduced MMP in rutin-treated HeLa cells

Mitochondrial membrane depolarization potential (ΔΨm) was analyzed in both untreated and rutin-treated HeLa cells with Mitotracker red stain. Figure 2A strongly presented the fact that rutin treatment resulted in significant reduction (in dose-dependent manner) in ΔΨ in HeLa cells which were represented by decrease in red fluorescence intensity. These findings indicated that this decrease in MMP could be one of the possible mechanisms behind apoptosis induction in rutin-treated HeLa cells.

### Rutin induced caspase activities in HeLa cancer cells

Caspases are family of cysteine proteases that trigger apoptosis via cleaving proteins at aspartic acid residues [18]. Thus, we investigated whether rutin-induced apoptosis in HeLa cancer cells was due to caspase activation. Significant induction of caspase-3, -8 and -9 activities were observed in rutin-treated HeLa cells after 24 h (Figure 2B). Figure 2B depicted significant increase in caspase-3, -8 and -9 activities in comparison with untreated (control) HeLa cells. Hence, a dose-dependent increase in caspase-3, -8 and -9 activities were reported in rutin-treated HeLa cells.

### Abrogation of rutin-induced apoptosis by caspase inhibitors

To illustrate whether rutin-induced cytotoxicity in HeLa cancer cells was associated with the activation of three caspases (caspase-3, -8 and -9), HeLa cells were pretreated with 50 µM of caspase-3, -8 and -9 inhibitors (Z-DEVD-FMK, Z-IETD-FMK and Z-LEHD-FMK) for 2 h and then treated with selective doses of rutin for 24 h. MTT assay was used to assess the cell viability as described above. Pretreatment with all the three caspase inhibitors potentially reduced the cytotoxicity in HeLa cancer cells caused by rutin treatment (Figure 2C). Altogether these findings strongly validated the crucial role of caspase activation in rutin-induced apoptosis.

### Rutin augmented the ROS level in HeLa cancer cells

**Figure 3:**
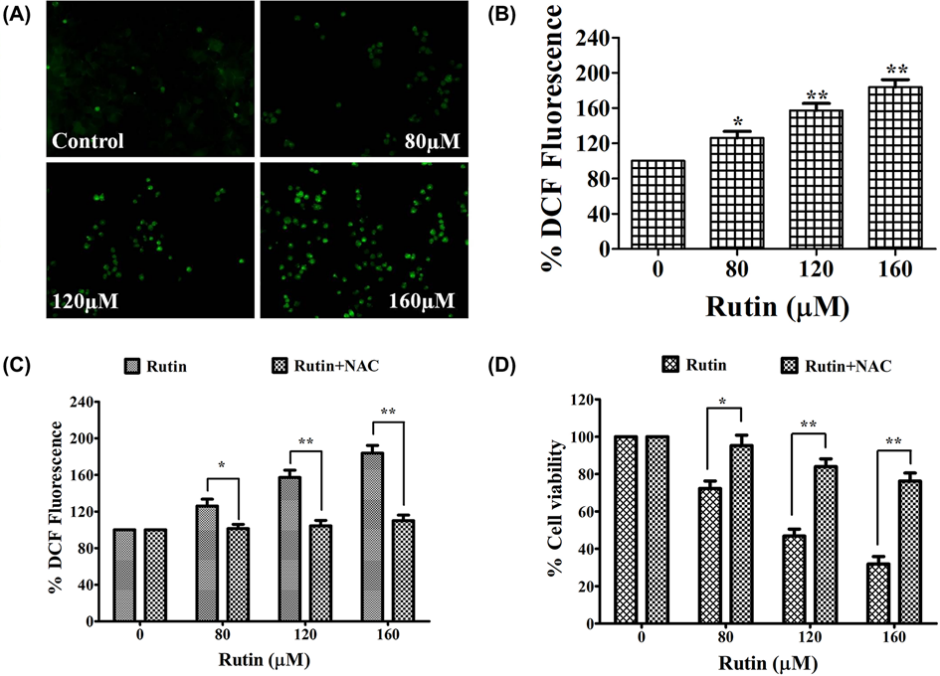
Increased ROS generation in rutin-treated HeLa cells (**A**) Increased ROS generation in DCFH-DA stained HeLa cancer cells exposed to selective doses of rutin observed by fluorescence microscopy. Presented data are representative of three independent experiments. (**B**) ROS quantification in terms of % DCFDA fluorescence in rutin-treated HeLa cells. (**C**) Percent cell viability of HeLa cells pretreated with NAC and then treated with rutin (0–160 μM) for 24 h estimated by MTT assay. (**D**) ROS level in HeLa cells preexposed with NAC (ROS inhibitor), and then treated with rutin. SEM and mean of three independent experiments were presented: *P<0.01, **P<0.001, in comparison to related control value.

To exhibit the involvement of ROS in cell growth inhibition and apoptosis induction in rutin-treated HeLa cancer cells, ROS production was investigated by employing fluorescence microscopy (CM-H2DCFDA fluorescent probe). Figure 3A clearly shows increased intracellular ROS level (significant fluorescence intensity) in rutin-treated cells for 12 h. Quantitative analysis also presented augmented ROS production in a dose-dependent manner (Figure 3B). Further, to corroborate the rutin-mediated augmentation of ROS level, HeLa cancer cells were pretreated with ROS inhibitor (NAC). Quantitative analysis displayed the attenuation of elevated ROS level in NAC (10 mM) pretreated HeLa cancer cells which strongly substantiated our research that rutin could enhance ROS level in HeLa cancer cells (Figure 3C).

### Abrogation of rutin-induced cytotoxicity by ROS inhibitor (NAC)

In order to establish the ROS involvement in rutin induced cytotoxicity in HeLa cancer cells, we investigated their effects in NAC (10 mM) pretreated HeLa cancer cells by MTT assay. Pretreated HeLa cells exhibited significant reduction in cytotoxicity caused by rutin (Figure 3D). Hence these findings clearly indicated that augmented ROS generation is crucial for rutin-induced apoptosis in Hela cancer cells.

### Rutin promoted G_0_/G_1_ phase cell cycle arrest in HeLa cancer cells

**Figure 4:**
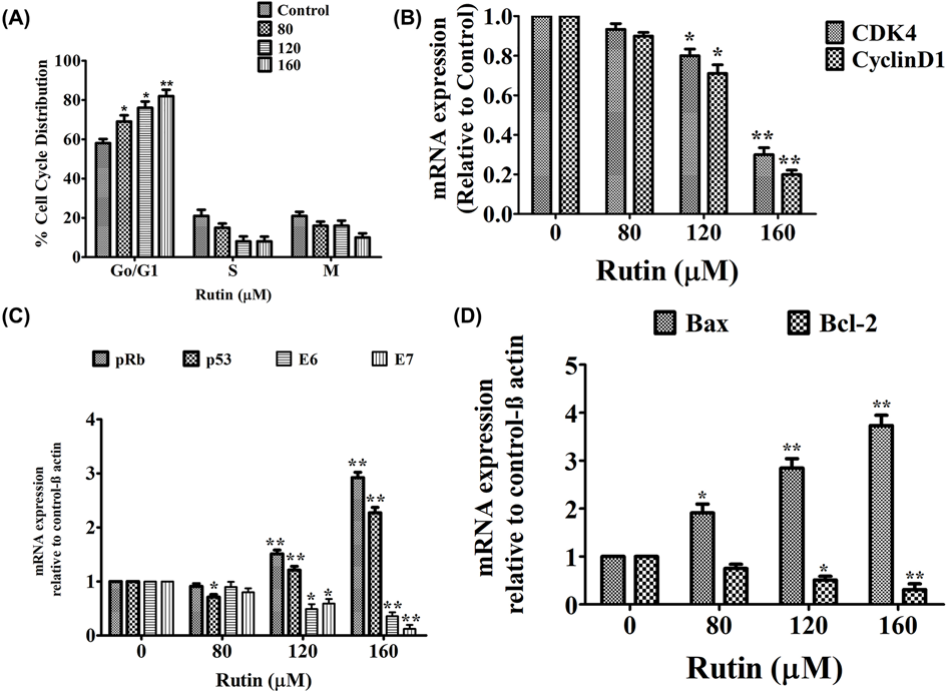
Effect of rutin on cell cycle modulation and mRNA expression of its associated genes (**A**) Cell cycle distribution in rutin-treated HeLa cancer cells after being exposed for 24 h. (**B**) Graph showing the expression level of cell cycle regulatory gene (*Cyclin D1* and *CDK4*) analyzed using RT-PCR (**C,D**) Graph showing the expression level of apoptotic genes (*p53, Bax, Bcl-2, pRb*) analyzed using RT-PCR SEM and mean of three independent experiments were presented: *P<0.01, **P<0.001, in comparison to related control value.

To explore the potential mechanism by which rutin impairs cancer cell proliferation, cell cycle progression was investigated by flow cytometer. Figure 4 depicted significant cell growth arrest at G_0_/G_1_ phase of cell cycle in rutin-treated HeLa cancer cells with increasing doses of rutin. In parallel, a collateral reduction in cells in S phase was observed whereas there was no significant changes were reported in G_2_/M population (Figure 4A). A significant reduction in CDK4 and cyclinD1 mRNA expression level was also observed in rutin-treated HeLa cells (Figure 4B).

### Effect of rutin on modulation of pRb, p53, Bax, Bcl2, E6 and E7 mRNA expression in HeLa cells

To explain the mechanism behind apoptosis in rutin-treated HeLa cancer cells, we inspected mRNA transcript level of apoptosis controlling genes by using RT-PCR. Rutin treatment significantly up-regulated the transcriptional level of tumor suppressors (p53 and pRb) in HeLa cells (Figure 4C). Rutin treatment also decreased the expression of Bcl-2 after 24 h of treatment. However, a significant increase was observed in the gene expression of Bax in rutin-treated cells (Figure 4D).

RT-PCR is used to explain how rutin might modulate E6 and E7 mRNA transcripts’ levels (cervical cancer oncogenes) that affects cell cycle progression. E6 and E7 mRNA expression were determined after 24 h of treatment of HeLa cells with rutin. Figure clearly depicted significant reduction in E7 and E6 mRNA expression level in rutin-treated HeLa cancer cells.

### Molecular interactions of rutin with E6 and E7 (HPV-18 oncoprotein) targets via docking analysis

**Figure 5:**
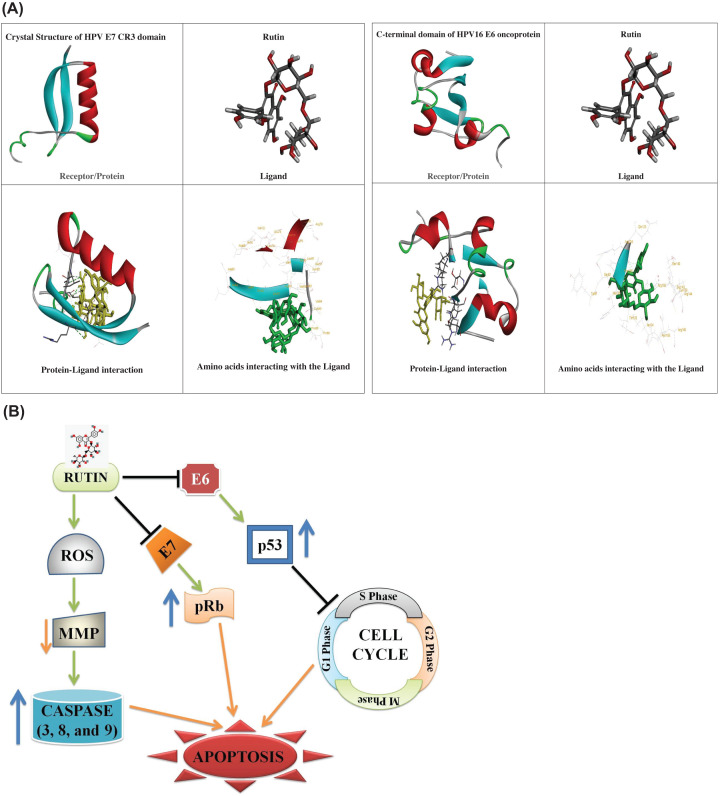
*In silico* analysis showing binding interaction between ligand and protein (**A**) *In silico* analysis of target (E6 and E7) and ligand (rutin) interaction with their binding interactions. (**B**) Possible mechanism behind the antiproliferative potential of rutin in HeLa cancer cells.

*In silico* findings also supported our findings by showing significant binding affinity for these two potent oncogenes E6 and E7 (Table 1). However, rutin has exhibited strong binding affinity for E7 in comparison to E6 protein. Figure 5 showed protein ligand interaction between E6 AND E7 and rutin. Altogether, these research findings pave us a strong way to further explore the pharmacokinetic parameters of rutin for its future use in drug designing.

**Table 1:** Molecular docking analysis of antiviral compounds against the structure of the C-terminal domain of HPV16 E6 oncoprotein (2LJZ)

Ligand	Target	Binding affinity (kcal/mol)	Amino acid residues
Rutin	C-terminal domain of HPV16 E6 oncoprotein (2LJZ)	3.54	Ser^82^, Tyr^81^, Phe^125^, Arg^124^, Gln^123^, His^126^, Thr^133^, Ile^104^, Asn^015^, Arg^146^, Arg^135^, Lys^139^, Ser^143^, Arg^144^, Ser^140^
	Crystal structure of HPV E7 CR3 domain (2B9D)	−2.21	Thr^89^, Pro^86^, Cys^85^, Val^84^, Ile^83^, Val^59^, Arg^60^, Leu^61^, Thr^62^, Val^63^, Leu^64^, Ala^65^, Ala^69^, Ile^70^, Gln^72^, Glu^74^, Leu^73^, Val^49^, Ala^50^, Leu^77^, Met^76^, Arg^79^, Leu^81^, Ser^51^, Asn^82^, Leu^78^

## Discussion

Rutin (glycosylated polyphenolic phytocompound) is found in several fruits and vegetables such as buckwheat, elderberry, japanese pagoda tree and has displayed significant anticancerous potential in numerous carcinomas including cervical, stomach, bladder, liver, prostate and lung cancers [36]. However, the molecular mechanisms behind its anticancer efficacies in cervical cancers are feebly understood. Major defects in cancer cells are either due to deregulated cell cycle or lack of control in cell growth and apoptosis [37]. Therefore this study was focused towards evaluating the anticancerous efficacy of rutin and analyzing the mechanisms associated with rutin-induced apoptosis in HeLa cancer cells. The cytotoxic efficacy against HeLa cancer cell lines was assessed following 24-h rutin treatment. HeLa cancer cells exhibited significant cytotoxicity at 24 h after rutin treatment without affecting normal cell proliferation (reported in our previous findings) [38]. In agreement with other published reports, in our study, rutin enhanced the inhibitory efficacy of 5-FU on the proliferation of HeLa cancer cells with less cytotoxicity in normal cells (HEK-293) [38].

Since the past few decades, a mainstay or foundation of clinical oncology (dealing with cancer malignancies) has been the elucidation and development of various therapies facilitating the effective removal of cancer or tumor cells by apoptosis. Moreover, this apoptosis (programmed cell death) process is mediated by numerous extrinsic or intrinsic signaling pathways triggered by several factors, such as cellular stress, immune surveillance and DNA damage. Several reports have shown strong correlation between apoptosis pathways and other signaling mechanisms in inducing cell death [39]. Apoptosis can be defined as one form of programmed cell death and well organized cellular suicide pathway [40]. Therefore this study was mainly focused on investigating the apoptosis potential of rutin and its association with apoptotic pathways in rutin-treated HeLa cells. Fragmented (or condensed) nucleus is an important hallmark of apoptotic induction. Our findings were supported by the above presented fact where fragmented (or condensed) nuclei in rutin-treated HeLa cells were observed with DAPI staining suggesting that rutin caused cell death via apoptosis. Markers of apoptotic activity and cytotoxic effects were induced by rutin treatment in HeLa cancer cells. Our study simultaneously elucidated the effects of rutin on genes involved in survival and apoptotic cellular in HeLa cancer cells. Rutin potentially induced the apoptosis of HeLa cancer cells via up-regulation of pro-apoptotic genes (p53 and Bax) and down-regulation of survival genes (including E7, E6 and Bcl-2). In addition, Bax/Bcl-2 ratio significantly increased in rutin-treated HeLa cells, indicating that rutin modulated mitochondrial function to arbitrate cell death.

Caspase activation is one of the crucial pathway associated with apoptosis induction that can be regulated by cytochrome *c*. Caspase-3 (an effector or executional caspase) recognizes and cleaves short amino acid sequences in various target proteins, leading to cell death (irreparable DNA damage) [41,42]. E6 and E7 hinder caspase-8 activation via procaspase-8 degradation [43]. Caspase-8 (molecular linker) is also known for bridging between intrinsic and extrinsic apoptosis pathway by cleaving Bid (Bcl-2 family) which further bind to Bax (a proapoptotic protein) resulting in release of cytochrome *c* (an initiator for mitochondrial pathway) [44]. In our study, rutin treatment resulted in up-regulated caspase-3, -9 and -8 expressions suggesting that rutin could induce apoptosis via mitochondria-mediated or extrinsic pathway. Moreover, rutin induced cytotoxicity in HeLa cells was remarkably reduced by all three caspase-3, -9 and -8 inhibitors, depicting crucial role of caspase-3 activation during rutin-induced apoptosis. Altogether these results strongly propose the proapoptotic role of rutin by inducing mitochondria-mediated pathway via activation of caspase-3, -8 and -9 on rutin-treated HeLa cells.

Mitochondria (cell powerhouse) play an important role in programmed cell death and maintenance of mitochondrial membrane integrity plays a significant role in cell survival. Several reports have suggested the association of mitochondrial dysfunction with cell apoptosis [45]. Usually, tumor cells have elevated MMP than normal epithelial cells. Hence, various anticancer agents such as phytocompounds can directly disturb mitochondrial respiration and glycolysis leading to an extensive ATP depletion, which then congregate with intrinsic apoptotic (death) pathway [46]. ROS are byproducts of cellular metabolism inside mitochondria and elevated ROS levels are often linked with DNA fragmentation, cellular damage and apoptosis [47]. Our data clearly indicate that rutin-induced apoptosis in HeLa cancer cells via ROS-mediated mitochondrial pathway.

It is well established fact that carcinogenesis is closely related to uncontrolled cell cycle [48]. Cell cycle analysis clearly exhibited that rutin arrested cell cycle progression of HeLa cancer cells in G_0_/G_1_ phase in corroboration with our previous findings [38]. Thus these results showed that rutin is not only responsible for apoptosis induction but also induced cell cycle arrest in HeLa cancer cells. Cell progression through several cell cycle phases (G_1_, S, G_2_ and M) is positively governed by cyclins [49]. In our findings, it can be seen that trutin treatment down-regulated the CDK4 and cyclin D1 mRNA expression (cell cycle markers). CDK4 and cyclin D1 mRNA expressions are down-regulated which could further explain cell cycle arrest in G_1_ phase. These findings were in accordance with previously published studies [38].

The inverse association between rutin and E6 and E7 mRNA expression in HeLa cancer cells has not yet been reported. E7 and E6 (oncoproteins of HPV) act cooperatively via interfering with several tumor suppressor proteins such as retinoblastoma protein (pRb), p53 and p27, respectively. HPV oncoproteins also evade apoptosis via suppressing caspase activation (key element in extrinsic and intrinsic apoptosis pathways). E6 is known to promote ubiquitin-dependent p53 degradation which is one of a crucial protein responsible for increased expression of cell cycle regulators needed for apoptosis. Meanwhile, E7 associate with hypophosphorylated form of pRb leading to E2F release from the complex (pRb–E2F). Subsequently this release leads to significant reduction in the expression of E7 and E6 in rutin-treated HeLa cells which further explain the mechanism behind restoration of p53 and pRB mRNA expression (at transcriptional level) in comparison to untreated HeLa cells. Hence, these modulatory effects (down-regulation of E6 and E7 mRNA) produced by rutin treatment could be the responsible factor for apoptotic induction in rutin-treated HeLa cells. Molecular docking further supported our *in vitro* findings by exhibiting strong binding affinity of rutin against E6 target in corroboration with *in vitro* findings. Although, rutin has not exhibited strong binding affinity against E6 protein in molecular docking analysis yet has significantly down-regulated E6 mRNA in HeLa cells. Altogether these research findings have strongly validated the potential of rutin as potent drug candidate against cervical cancer via targeting E6 and E7 genes. In summary, our findings strongly established that rutin exhibited apoptotic efficacy against HeLa cancer cells via caspase activation, MMP disruption, ROS augmentation and cell cycle arrest at G_0_/G_1_ phase (Figure 5B).

## Conclusion

E6 and E7 (HPV oncoplayers) are the major driving force for the progression of cervical cancer and play important role in carcinogenesis right from the beginning such as maintenance of proliferative signaling, escape of tumor suppressor, telomerase activation, angiogenesis induction and metastasis. Our findings strongly demonstrated that rutin could induce apoptosis in dose-dependent manner via inhibiting of E6 and E7 mRNA expression and increasing pRb, Bax/Bcl-2 and p53 in HeLa cancer cells, while posing minimal toxic effects on normal cells. Altogether, rutin has strong potential in chemoprevention due to its low cost, safety and bioavailability. On the other side, down-regulation or inhibition of E6 and E7 expression by rutin could provide an effective and safe therapeutic approach for cervical cancer.

## Data Availability

All data are available from the corresponding author on reasonable request.

## Abbreviations

5-FU: 5-fluorouracil
DAPI: 4′,6-diamidino-2-phenylindole
DCFH-DA: 2′,7′-dichlorodihydrofluorescein diacetate
DMEM: Dulbecco’s modified Eagle’s medium
FBS: fetal bovine serum
HPV: human papillomavirus
LGA: Lamarckian Genetic Algorithm
MMP: mitochondrial membrane potential
MTT: 3-(4,5-dimethylthiazol-2-yl)-2,5-diphenyl tetrazolium bromide
NAC: N-acetyl-l-cysteine
PBS: phosphate buffer saline
ROS: reactive oxygen species
